# CEO Overconfidence, Corporate Governance, and R&D Smoothing in Technology-Based Entrepreneurial Firms

**DOI:** 10.3389/fpsyg.2022.944117

**Published:** 2022-07-13

**Authors:** Yu Huang, Xinchun Wang, Yuanqin Li, Xiaoyu Yu

**Affiliations:** ^1^School of Management, Shanghai University, Shanghai, China; ^2^John Chambers College of Business and Economics, West Virginia University, Morgantown, WV, United States

**Keywords:** CEO overconfidence, R&D smoothing, intertemporal decision-making, internal control, institutional investors, upper echelons theory, technology-based entrepreneurial firm, uncertainty in entrepreneurship

## Abstract

The intertemporal stability of research and development (R&D) investment is a key issue in successfully promoting the continuation of innovation activities under high uncertainty in entrepreneurship. R&D smoothing helps firms to navigate the uncertainties of the external environment and maintain the stability of their investments in innovation. Chief executive officers (CEOs) are the most important decision-makers in firms' strategic planning. However, overconfident CEOs may overlook the importance of their firms' strategic actions on innovative activities. Drawing on upper echelons theory, this paper examines how CEO overconfidence affects firms' R&D smoothing. Using a sample of firms listed in China's Growth Enterprises Market between 2013 and 2020, this study finds that CEO overconfidence has a significant negative impact on R&D smoothing. Furthermore, our findings reveal that firms' internal control quality and institutional investor monitoring can mitigate the negative association between CEO overconfidence and R&D smoothing. Our findings provide new insights into the micro-level theoretical explanations for R&D smoothing and offer practical implications for technology-based entrepreneurial firms.

## Introduction

As an important innovative activity, research and development (R&D) requires continuous investment (Hall, 2002). Sudden interruptions and the need to restart R&D investments may result in large financial losses (Himmelberg and Petersen, 1994). To succeed in R&D activities, firms must avoid unexpected R&D adjustments (Brown and Petersen, 2011) and smoothen the path for long-term R&D investment. During the Nasdaq Stock Market's volatile period from 1998 to 2002, young firms actively engaging in R&D activities generally used their cash reserves to dampen the volatility in R&D investment by ~75% (Brown and Petersen, 2011). This strategic action is often referred to as R&D smoothing—that is, maintaining the stability of R&D investments over time through establishing and using preventive cash reserves (Brown and Petersen, 2011). R&D smoothing is an important coping strategy for firms when faced with an uncertain external environment. Furthermore, R&D smoothing helps firms to maintain the stability of their investments in innovative activities, particularly in R&D-intensive firms, such as technology-based entrepreneurial firms (TBEFs), which usually have both intelligence- and capital-intensive features. However, TBEFs are often at a disadvantage in terms of market resource competition (Yu and Wang, 2021 and face higher external environmental uncertainty than mature firms. Therefore, R&D smoothing is as a key strategic action for TBEFs to establish strategic competitive advantages.

Previous studies have examined various factors affecting R&D smoothing, including financing constraints (Brown and Petersen, 2011), market timing (Shin and Kim, 2011), and innovation efficiency (Liu et al., 2021). However, these studies lack insight into the micro-foundations of R&D smoothing. More specifically, the literature on the determinants of R&D smoothing mainly focuses on organizational and contingent factors, and individual-level factors are not well-understood. In particular, firms' chief executive officers (CEOs) are their most important decision-makers; thus, CEOs' personal attributes might have a substantial impact on their firms' strategic activities (Lin et al., 2020; Jia et al., 2021; Yu et al., 2022c), which motivates a deeper exploration of CEO-level factors.

Upper echelons theory posits that executives' personal characteristics largely determine organizational decisions (Hambrick and Mason, 1984). Accordingly, TBEF CEOs exhibit high levels of overconfidence (Forbes, 2005) because they tend to have relatively high subjective estimates of their own abilities, judgments, or prospects (Hirshleifer et al., 2012). As a result, overconfident CEOs may overlook the importance of ensuring stable innovation investment when experiencing high environmental uncertainty. In this study, we examine whether and how CEO overconfidence affects TBEFs' R&D smoothing behavior.

In addition, corporate governance may influence CEOs' decision-making behavior (Munari et al., 2010; Chrisman and Patel, 2012; Hoitash and Mkrtchyan, 2022). By increasing principals' decision-making participation, corporate governance can reduce CEOs' excessive control over innovation investment decisions, thereby correcting CEO overconfidence. Therefore, we examine the moderating effect of corporate governance on the relationship between CEO overconfidence and R&D smoothing. Specifically, to incorporate an internal governance perspective, we examine a firm's internal control quality. Internal control refers to the various control and adjustment plans, organizational mechanisms, procedures, and methods implemented within an firm to effectively obtain and use various resources, improve operating efficiency, and achieve established management goals. We posit that high-quality internal controls can monitor and regulate the behaviors of overconfident CEOs. Furthermore, to incorporate external governance perspectives, we examine the moderating effect of the level of monitoring provided by institutional investors. Institutional investor monitoring refers to investors' active exercise of power by participating in corporate governance to supervise and influence executives' decisions. We posit that high levels of institutional investor monitoring can shape overconfident CEOs' decision-making behavior to mitigate the influence of CEO overconfidence on R&D smoothing.

We offer three contributions to the literature. First, by investigating the impact of CEO overconfidence, we provide a new perspective for understanding TBEFs' decisions to engage in R&D smoothing and increase our understanding of the micro-foundations of R&D smoothing behavior. This finding also provides new insights into the economic consequences of CEO overconfidence. Although the literature offers some preliminary insights into the effects of CEO overconfidence on innovation, these studies are limited to the static characteristics of R&D investment. We emphasize that R&D investment is a dynamic process in which CEO characteristics, such as overconfidence, affect R&D smoothing. Second, the literature overlooks the influence of internal controls on overconfident CEOs' intertemporal decision-making behavior. We improve our understanding of R&D smoothing by investigating the moderating effect of internal control quality on shaping overconfident CEOs' intertemporal R&D investment behavior. Third, we add to the literature concerning the governance role of institutional investors. Specifically, we confirm that institutional investors have a role in monitoring CEOs and guiding their appropriate decision-making related to R&D smoothing.

## Literature Review and Research Hypothesis

### Relationship Between CEO Overconfidence and R&D Smoothing

Upper echelons theory (Hambrick and Mason, 1984) suggests that CEOs' cognitive characteristics can affect firms' strategic decision-making behavior (Nielsen, 2010). Overconfidence refers to individuals' tendency to make relatively subjective estimates of their abilities, judgments, or prospects (Hirshleifer et al., 2012). In other words, it relates to individuals' overestimation of their ability to produce good results (Weinstein, 1980; Alicke, 1985). This phenomenon is especially prominent among senior executives (Langer, 1975; Larwood and Whittaker, 1977; Cooper et al., 1988). Overconfidence results in CEOs overestimating their knowledge and abilities, underestimating risks, and overestimating their ability to control events (Nofsinger, 2005), with a significant effect on their R&D investment decisions (Simon and Houghton, 2003; Galasso and Simcoe, 2011). In particular, CEO overconfidence will likely increase R&D investment in innovation (Galasso and Simcoe, 2011; Hirshleifer et al., 2012). We posit that overconfident CEOs will engage in less R&D smoothing for two reasons.

First, overconfident CEOs of TBEFs tend to overestimate their ability to control future uncertainty. As a result, they tend to reduce their firms' R&D smoothing behavior. Moreover, overconfident CEOs tend to believe that they are equipped to accurately predict future trends; therefore, they may also believe that they are able to control and mitigate future adverse events (March and Shapira, 1987). As a result, overconfident CEOs tend to believe that they can both manage R&D projects that are short of funds and successfully manage any potential future funding issues that may emerge during periods of financial difficulty. As such, overconfident CEOs are often reluctant to respond future adverse events by keeping sufficient cash reserves (Huang et al., 2016).

Second, overconfident CEOs of TBEFs tend to overestimate the positive future prospects of their firm R&D activities and therefore tend to engage in reduced levels of R&D smoothing. Overconfident CEOs also tend to overestimate the likelihood of success and underestimate the possibility of failure (Larwood and Whittaker, 1977), overestimate the expected benefits of a given project (Schrand and Zechman, 2012), and underestimate the foreseeable cost of the project (Schiff and Lewin, 1970). These actions often lead to adverse volatility in R&D investment during the R&D cycle, thereby reducing the stability of TBEFs' R&D expenditures. Overconfident CEOs also often underestimate the likelihood of financial shocks during their R&D activities and underestimate the possibility of changes in the external environment. Thus, overconfident CEOs tend to avoid keeping sufficient cash reserves to maintain the long-term stability of their R&D investments. Therefore, we propose the following hypothesis:

Hypothesis 1. Overconfident CEOs will weaken the level of R&D smoothing of TBEFs.

### Moderating Effect of Internal Control Quality

Internal governance is important for innovative decision-making behavior (Chrisman and Patel, 2012) and internal governance mechanisms can influence overconfident CEOs' decision-making behavior (Anand and Anjan, 2008); therefore, internal control is one of the most important internal governance mechanisms available to TBEFs. Internal control refers to the various control and adjustment plans, procedures, and methods implemented within an organization to effectively obtain and use various resources, improve operating efficiency, and achieve established management goals. Organizations engage in internal control practices to ensure reasonable business management, legal compliance, and asset security, improve the efficiency and effectiveness of business operations, promote the realization of development strategies, and ensure that financial reports and related information are truthful and complete. Several studies assess the influence of internal control by boards of directors on CEO overconfidence using a variety of variables, including the proportion of independent directors to company directors (Mohamed et al., 2012). The quality of internal control has a negative moderating effect on mergers and acquisitions by overconfident CEOs (Kolasinski and Li, 2013). As such, we posit that when internal control quality is high in TBEFs, the weakening effects of overconfidence on R&D smoothing will be reduced. We elucidate the rationale behind our theory below.

First, TBEFs with high-quality internal control usually use a scientific decision-making process to restrict CEOs' decision-making power (Banerjee et al., 2015) and reduce overconfident CEOs' tendency to make decisions based on their subjective judgment. These aspects will alleviate any negative impact on R&D smoothing from CEO overconfidence. More specifically, by establishing a scientific decision-making mechanism, high-quality internal control practices encourage CEOs to exercise caution when making decisions (and also repeatedly evaluate the potential consequences of their decisions) in addition to strengthening the engagement of internal and external stakeholders in decision-making processes. In this scenario, both sets of stakeholders can supervise decision-making and implementation processes. Increasing principals' decision-making participation reduces overconfident CEOs' excessive control over R&D smoothing decisions, suppresses the CEOs' illusion of control, and addresses CEOs' cognitive biases to alleviate the negative effects of CEO overconfidence on firms' R&D smoothing behavior.

Second, high-quality internal control results in better risk assessment, response procedures, and other risk management systems, as well as review procedures and authorization approvals, among other internal control activities. Improving these mechanisms can effectively prevent overconfident CEOs from overestimating positive future results, which encourages CEOs' preventive behavior. The effective use of these mechanisms can alleviate the negative effects of CEOs' overconfidence on firms' R&D smoothing behavior. High-quality internal control can effectively control and reduce corporate risk (Ashbaugh-Skaife et al., 2009), help companies set goals, ensure the continuity and repeatability of R&D project risk assessment activities, and identify and evaluate various potential risks in a timely fashion, which helps companies achieve their innovative goals. High-quality internal control encourages CEOs of TBEFs to pragmatically evaluate future costs, project benefits, and financial risks; therefore, internal control enable overconfident CEOs to more reasonably predict the future prospects for their R&D activities. These actions can alleviate the negative effects of CEO overconfidence on R&D smoothing. Therefore, we propose the following hypothesis:

H2. Compared with low-quality internal control practices, high-quality internal control decreases the weakening effect of overconfident CEOs on R&D smoothing.

### Moderating Effect of Institutional Investor Monitoring

Institutional investors play an important monitoring role in influencing CEOs' innovation decisions (Shleifer and Vishny, 1986; Munari et al., 2010). The monitoring role of institutional investors is an increasingly important external corporate governance mechanism for TBEFs. Institutional investors participate in corporate governance activities by actively exercising power, supervising and influencing management decision-making behavior, and urging companies to improve their operating performance. As firm owners, institutional investors have strong incentives to monitor their executive's behavior and take actions that increase their firm's value (Kang et al., 2018). Institutional investors can influence a company's decision-making behavior in a variety of areas, including mergers and acquisitions (Ferreira et al., 2010), payment policy (Grinstein and Michaely, 2005), executive compensation (Hartzell and Starks, 2003), earnings management (Chung et al., 2002), and hedging policy (Tai et al., 2014). We posit that high institutional investor monitoring in TBEFs reduces the weakening effects of CEO overconfidence on R&D smoothing for two reasons.

First, in TBEFs with high institutional investor monitoring, institutional investors will be more sensitive to risk because of their familiarity with the particularities of R&D activities. As a result, they will encourage firms to fully consider implementing preventive measures to anticipate future uncertainties. As such, institutional investors will negotiate with management and make recommendations to mitigate overconfident CEOs' subjective behavior (Smith, 1996; Carleton et al., 1998; Gillan and Starks, 2000), such as overconfident CEOs overestimating the positive future prospects of their R&D activities. In this way, institutional investors will influence CEOs' decisions and prevent overconfident CEOs from reducing R&D smoothing levels.

Second, the loss of institutional investors (usually major company shareholders) in TBEFs with high levels of institutional investor monitoring will result in certain checks and balances on CEOs' irrational decisions (McCahery et al., 2016). Indeed, CEOs' overtly irrational behavior will usually lead to dissatisfaction among institutional investors and prompt them to sell their shares (Parrino et al., 2003), which affects long-term development. Given this risk, CEOs may be more cautious in considering their ability to control the uncertainty of their future R&D activities and avoid irrational decisions that would prevent the smooth progress of their enterprise's R&D activities. In other words, the threat of losing institutional investors can prevent the negative effects of CEO overconfidence on R&D smoothing behavior. Therefore, we propose the following hypothesis:

H3. Compared with low institutional investor monitoring, high institutional investor monitoring decreases the weakening effect of overconfident CEOs on R&D smoothing.

## Materials and Methods

### Sample Selection and Data Resources

TBEFs commonly conduct R&D smoothing to protect their R&D investments. To assess the effects of the micro-level factor of CEO overconfidence on the varying levels of R&D smoothing across different companies and whether existing internal and external governance mechanisms can restrain overconfident CEOs' R&D smoothing behavior, we use a sample of firms listed in China's Growth Enterprises Market (or Second-Board Market) between 2013 and 2020. We choose these firms for three reasons. First, firms listed on the Growth Enterprises Market are mainly TBEFs. Second, the independent innovation of firms in the Growth Enterprises Market is mainly characterized by a longer R&D cycle and higher uncertainty. Third, the R&D investment in the model must take the previous year's data. However, the data for R&D investment before 2013 are missing too much information; therefore, we use 2013 as the starting year for our sample.

We obtain our data from the China Stock Market & Accounting Research and WIND databases and the China Listed Firms' Internal Control Index by Shenzhen Dibo Enterprise Risk Management Technology Co., Ltd. We deal with data in the following steps: (1) we exclude companies labeled as ST (i.e., those that have suffered losses for two consecutive years) and ^*^ST (i.e., those that have suffered losses for three consecutive years); (2) we exclude listed companies from the financial industry; (3) we exclude samples from the initial public offering (IPO) year to eliminate the impact of IPO; and (4) because we adopt a systemic generalized method of moments (GMM) estimation, which requires the construction of balanced panel data, we follow Brown and Petersen (2011) and exclude samples for which the main variable is missing in a given sample period. After completing our screening, we select 195 companies with a total of 1,365 observations as the research sample. We use Stata software (v. 15.0; Stata Corp, College Station, TX, USA) to process the data. Our sample size is relatively large; therefore, we winsorize all of the continuous variables at the bottom and top 1% levels to reduce the influence of outliers.

### Variable Measurement

#### R&D Smoothing

Considering the dynamic characteristics of R&D investment, we follow Brown and Petersen (2011) and use the correlation coefficient between the change in cash holdings and R&D investment to measure R&D smoothing.


(1) $$\begin{array}{r} RD_{i;t}=\beta_{0}+\beta_{1}\text{Δ}Cash_{i;t}+\beta_{2}RD_{i;t-1}+\beta_{3}{\left(RD_{i;t-1}\right)}^{2}\;\\ \;+\beta_{4}Stki_{i;t}+\beta_{5}Stki_{i;t-1}+\beta_{6}Loan_{i;t}+\beta_{7}Loan_{i;t-1}\;\\ \;+\beta_{8}CF_{i;t}+\beta_{9}CF_{i;t-1}+\beta_{10}Growth_{i;t-1}+\beta_{11}Q_{i;t-1}\;\\ \;+\beta_{12}Size_{i;t-1}+\beta_{13}Age_{i;t-1}+Ind_{i}+Year_{t}+\alpha_{i}\;\\ \;+\eta_{t}+\varepsilon_{i;t} \end{array}$$

where *RD*_*i*;*t*_ represents R&D expenditure and Δ*Cash*_*i*;*t*_ represents changes in cash holdings in Equation (1). For firms actively using cash holdings to smooth R&D, if the change in cash holdings (Δ*Cash*) is included with other sources of finance in an R&D regression, it will attract a negative coefficient since (holding other sources of finance constant) cash holding reductions free liquidity for R&D and cash holding increases decrease liquidity (Brown and Petersen, 2011). In other words, if firms use cash holdings to smooth R&D, the coefficient β_1_ of the change in cash holdings should be significantly negative. If firms do not engage in this practice, the coefficient β_1_ should be approximately zero.

#### CEO Overconfidence

Four methods can be used to measure CEO overconfidence: (1) stock option-based measure (Malmendier and Yan, 2011); (2) press-based measure (Malmendier and Yan, 2011); (3) management earnings forecasts bias (Lin et al., 2005); and (4) CEOs' relative salary (Hayward and Hambrick, 1997). First, stock option incentives are not implemented widely in China, and the corresponding data cannot be obtained (Firth et al., 2006). Second, scholars widely use financial press-based measure of CEO overconfidence, but because of the lack of relevant databases in China, the operability of this method is relatively low. Third, listed firms in China rarely make performance forecasts in advance. Most earnings forecasts are released near the time of performance disclosure; hence, there is a certain deviation in this measurement indicator. Fourth, considering the availability of data and the actual situation of China's securities market, we follow Hayward and Hambrick (1997) in using the CEOs' relative salary to measure their overconfidence. The higher the CEOs' salary relative to other executives, the more likely CEOs are to be overconfident (Hayward and Hambrick, 1997) and to exercise more power (Brown and Sarma, 2007). More specifically, Hayward and Hambrick (1997) use the ratio of the first-highest salaries divided by the second-highest salaries among executives to measure CEO overconfidence. However, because only the sum of the top three executives with the highest salaries and the sum of the salaries of all executives are disclosed in the accounting reports of listed firms of China, we use the ratio of the sum of the salaries of the top three executives with the highest salaries divided by the sum of the salaries of all executives as a proxy for CEO overconfidence. The higher the ratio, the higher the level of CEO overconfidence.

#### Internal Control Quality

We select the China Listed Firms' Internal Control Index as the proxy variable of internal control quality. The value range of the index is from 0 to 1,000, where larger values represent higher levels of internal control quality. Considering the value range of this index, we construct dummy variables to make the results easier to analyze. Specifically, if the index is greater than the industry annual median, the value of internal control quality is 1, indicating that the TBEF has a relatively high level of internal control quality, and 0 otherwise.

#### Institutional Investor Monitoring

We follow Tee (2020) and adopt the institutional investor's shareholding ratio as a proxy variable for the level of institutional investor monitoring, where higher ratios represent higher levels.

#### Control Variables

In terms of the control variables, we use lagged R&D (*RD*_*i*;*t*−1_) to consider the effect of the previous period's R&D investment on current R&D investment, and the quadratic term (${\left(RD_{i;t-1}\right)}^{2}$) relates to the adjustment cost of R&D investment (Bond and Meghir, 1994). We control for other major sources of R&D funding, including equity financing (*Stki*), debt financing (*Loan*), and cash flow (*CF*). We also control overconfidence (*OC*) because it is likely to affect R&D investment (Hirshleifer et al., 2012). We also use the growth rate of operating income (*Growth*) and market-to-book ratio (*Q*) as control variables for investment demand. In addition, we control for the size (*Size*) and age (*Age*) of the firms and the industry (*Ind*_*i*_) and year (*Year*_*t*_) effects. The model includes firm- (α_*i*_) and time-specific (η_*t*_) effects. In addition, *i* and *t* represent the firm and year, respectively, and ε_*i*;*t*_ represents the random error term.

#### Empirical Model

We follow Brown and Petersen (2011) and Bond and Meghir (1994) in establishing Equation (2) to test *H1*, based on the theoretical analysis considering the various factors affecting R&D investment:


(2) $$\begin{array}{l} RD_{i;t}=\beta_{0}+\beta_{1}\text{Δ}Cash_{i;t}+\beta_{2}{\left(OC\times \text{Δ}Cash\right)}_{i;t}+\beta_{3}OC_{i;t}\;\\ \;+\beta_{4}RD_{i;t-1}+\beta_{5}{\left(RD_{i;t-1}\right)}^{2}+\beta_{6}Stki_{i;t}+\beta_{7}Stki_{i;t-1}\;\\ \;+\beta_{8}Loan_{i;t}+\beta_{9}Loan_{i;t-1}+\beta_{10}CF_{i;t}+\beta_{11}CF_{i;t-1}\;\\ \;+\beta_{12}Growth_{i;t-1}+\beta_{13}Q_{i;t-1}+\beta_{14}Size_{i;t-1}\;\\ \;+\beta_{15}Age_{i;t-1}+Ind_{i}+Year_{t}+\alpha_{i}+\eta_{t}+\varepsilon_{i;t} \end{array}$$

where *OC*_*i*;*t*_ represents overconfidence. In line with *H1*, we mainly focus on the coefficient of *OC* × Δ *Cash*_*i*;*t*_. If the regression coefficient β_2_ is significantly positive, it indicates that an overconfident CEO will weaken R&D smoothing.

Based on Equation 2, we build Equation (3) to test *H2* regarding the moderating effect of internal control quality on the relationship between CEO overconfidence and R&D smoothing.


(3) $$\begin{array}{l} RD_{i;t}=\beta_{0}+\beta_{1}\text{Δ}Cash_{i;t}+\beta_{2}{\left(OC\times \text{Δ}Cash\right)}_{i;t}\;\\ \;+\beta_{3}{\left(Ind\times OC\times \text{Δ}Cash\right)}_{i;t}+\beta_{4}{\left(Ind\times \text{Δ}Cash\right)}_{i;t}\;\\ \;+\beta_{5}OC_{i;t}+\beta_{6}Ind_{i;t}+\beta_{7}RD_{i;t-1}+\beta_{8}{\left(RD_{i;t-1}\right)}^{2}\;\\ \;+\beta_{9}Stki_{i;t}+\beta_{10}Stki_{i;t-1}+\beta_{11}Loan_{i;t}+\beta_{12}Loan_{i;t-1}\;\\ \;+\beta_{13}CF_{i;t}+\beta_{14}CF_{i;t-1}+\beta_{15}Growth_{i;t-1}+\beta_{16}Q_{i;t-1}\;\\ \;+\beta_{17}Size_{i;t-1}+\beta_{18}Age_{i;t-1}+Ind_{i}+Year_{t}+\alpha_{i}\;\\ \;+\eta_{t}+\varepsilon_{i;t} \end{array}$$

where *Ind*_*i*;*t*_ represents internal control quality. In line with *H2*, we mainly focus on the coefficient of *Ind* × *OC* × Δ*Cash*_*i*;*t*_. If the regression coefficient β_3_ is significantly negative, this indicates that internal control quality negatively regulates the weakening effect of CEO overconfidence on R&D smoothing.

Based on Equation 3, we build Equation (4) to test *H3* regarding the moderating effect of the level of institutional investor monitoring on the relationship between CEO overconfidence and R&D smoothing.


(4) $$\begin{array}{l} RD_{i;t}=\beta_{0}+\beta_{1}\text{Δ}Cash_{i;t}+\beta_{2}{\left(OC\times \text{Δ}Cash\right)}_{i;t}\;\\ \;+\beta_{3}{\left(Pro\times OC\times \text{Δ}Cash\right)}_{i;t}+\beta_{4}{\left(Pro\times \text{Δ}Cash\right)}_{i;t}\;\\ \;+\beta_{5}OC_{i;t}+\beta_{6}Pro_{i;t}+\beta_{7}RD_{i;t-1}+\beta_{8}{\left(RD_{i;t-1}\right)}^{2}\;\\ \;+\beta_{9}Stki_{i;t}+\beta_{10}Stki_{i;t-1}+\beta_{11}Loan_{i;t}+\beta_{12}Loan_{i;t-1}\;\\ \;+\beta_{13}CF_{i;t}+\beta_{14}CF_{i;t-1}+\beta_{15}Growth_{i;t-1}+\beta_{16}Q_{i;t-1}\;\\ \;+\beta_{17}Size_{i;t-1}+\beta_{18}Age_{i;t-1}+Ind_{i}+Year_{t}+\alpha_{i}\;\\ \;+\eta_{t}+\varepsilon_{i;t} \end{array}$$

where *Pro*_*i*;*t*_ represents the level of institutional investor monitoring. In line with *H3*, we mainly focus on the coefficient of *Pro* × *OC* × Δ*Cash*_*i*;*t*_. If the regression coefficient β_3_ is significantly negative, it indicates that the level of institutional investor monitoring reduces the weakening effect of CEO overconfidence on R&D smoothing.

#### Estimation Method

Our models are dynamic panel regression models because they include the lag term of R&D expenditure. However, the explanatory variables may feature endogeneity problems. Arellano and Bover (1995) and Blundell and Bond (1998) suggest that a systemic GMM estimation should be used to overcome the above two problems. This method has two advantages: (1) instrumental variables or differences can be used to control the unobservable firm- and time-specific effects and (2) the explanatory and explained variables of the lag period can be used as instrumental variables to overcome the model's endogeneity problem.

We estimate our models using one-step systemic GMM estimation. We follow Brown and Petersen (2011) and treat all financial variables (including Δ*Cash, Stki, Loan*, and *CF*) as potentially endogenous, and we use lagged levels dated *t*−*2* and *t*−*4* as instruments for the regression in differences and lagged differences dated *t*−*1* for the regression in levels. Furthermore, we follow Arellano and Bond (1991) and report the results of serial correlation and overidentification (i.e., Sargan) tests to evaluate the effectiveness of the instrumental variables. In addition, we decentralize the cross terms involved in the model to prevent multicollinearity.

## Results

### Descriptive Statistics

**Figure 1:**
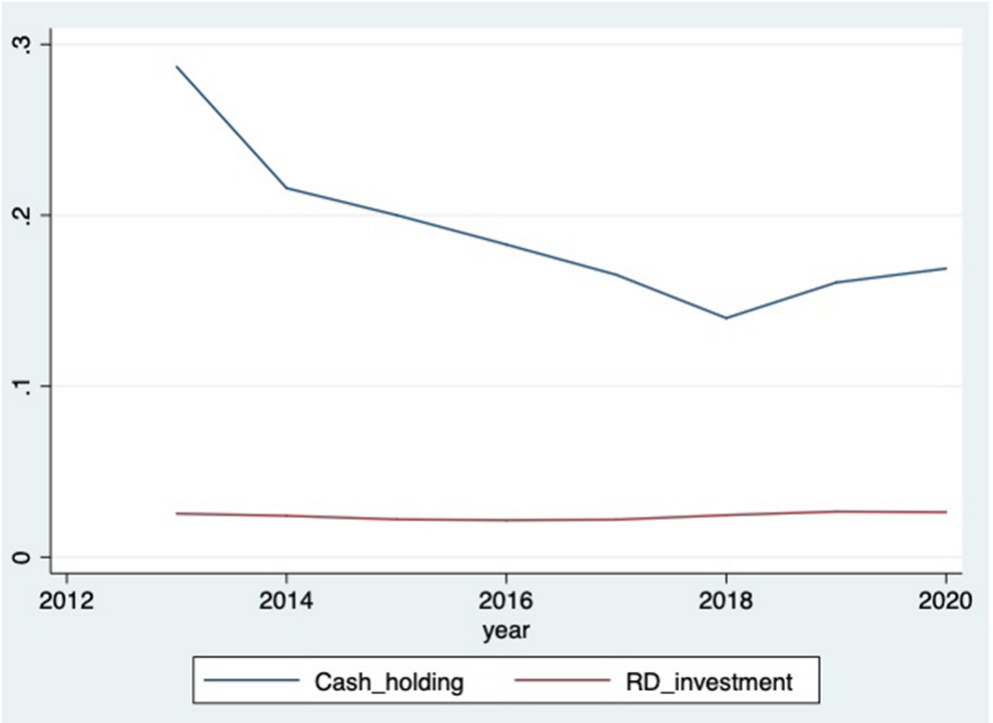
TBEFs' R&D investment and cash holdings. This figure plots the total R&D investment and cash holdings to the total assets ratios of TBEFs in our sample. R&D investment shows a more stable trend from 2013 to 2020.

Table 1 lists the results of the descriptive statistical analysis of the main variables. The standard deviation is 0.020 and the maximum value is 0.105, indicating that there are large differences in R&D investment activities between the different firms. The lowest value of changes in cash holdings *(*Δ*Cash*) is −0.265 and the highest value is 0.439, indicating that there are strong variability and inherent differences in cash holdings. These results confirm that firms use changes in cash holdings to smooth R&D investment. As shown in Figure 1, R&D investment is far smoother than cash holdings from 2013 to 2020. The average ratio of the sum of the salaries of the top three executives with the highest salaries divided by the sum of the salaries of all executives (*OC*) is 0.436, indicating that CEOs of TBEFs have a higher level of overconfidence.

**Table 1:** Descriptive statistics.

**Variables**	**Mean**	**Standard deviation**	**Minimum**	**Maximum**
*RD*	0.027	0.020	0.000	0.105
Δ*Cash*	0.019	0.110	−0.265	0.439
*OC*	0.436	0.113	0.220	0.780
*Ind*	0.482	0.500	0.000	1.000
*Pro*	0.281	0.203	0.001	0.765
*Stki*	0.028	0.067	0.000	0.338
*Loan*	0.137	0.149	0.000	0.742
*CF*	0.040	0.060	−0.129	0.216
*Growth*	0.202	0.399	−0.523	2.117
*Size*	21.755	0.805	20.090	23.884
*Age*	15.900	4.553	5.000	28.000
*Q*	2.573	1.466	0.994	8.735

*This table presents the descriptive statistics of the main variables, which are defined as R&D investment (RD), change in cash holding (ΔCash), CEO overconfidence (OC), internal control quality (Ind), institutional investor monitoring (Pro), equity financing (Stki), debt financing (Loan), cash flow (CF), growth rate of operating revenue (Growth), firm size (Size), firm age (Age) and market-to-book ratio (Q). There are 1,356 sample observations*.

### Hypothesis Test Analysis

We perform serial correlation and over-identification tests on our three main models. The Arellano–Bond first- and second-order serial correlation tests show that the *p* value of the *M1* statistic for all of the models is 0 (*p* < 0.05), and the *p* values of the *M2* statistic are 0.986, 0.898, 0.719 and 0.787 for Equations (1) to (4), respectively (*p* > 0.05), indicating that there is only a first-order serial correlation in the perturbation term but no second-order serial correlation, confirming the null hypothesis that the disturbance term has no autocorrelation. These results indicate that the systemic GMM estimation is used correctly. The results of the overidentification test show that the *p* values are 0.158, 0.145, 0.211, and 0.221 (*p* > 0.05) for Equations (1) to (4), respectively, indicating that our hypothesis (i.e., all instrumental variables are valid) cannot be rejected at the 5% significance level. Therefore, there are no overidentification problems in the four models in this study.

As shown in Table 2, the regression results for Equation (1) demonstrate that the estimated coefficient of changes in cash holdings Δ*Cash* β_1_ is −0.024 (*z* = −1.70, *p* < 0.1), indicating a significant negative correlation between changes in cash holdings and R&D investment. In other words, TBEFs use cash holdings to smooth R&D investment.

**Table 2:** Test results for the effects of CEO overconfidence on R&D smoothing and the moderating effects of internal and external corporate governance.

**Variables**	**Equation (1)**	**Equation (2)**	**Equation (3)**	**Equation (4)**
Δ*Cash_*t*_*	−0.024^*^	−0.118^**^	−0.224^*^	−0.189^*^
	(−1.70)	(−1.98)	(−1.73)	(−1.80)
*OC × ΔCash_*t*_*		0.256^*^	0.480^*^	0.404^*^
		(1.92)	(1.70)	(1.67)
*Ind × OC × ΔCash_*t*_*			−0.527^*^	
			(−1.78)	
*Pro × OC × ΔCash_*t*_*				−1.003^*^
				(−1.90)
*Ind × ΔCash_*t*_*			0.246^*^	
			(1.80)	
*Pro × ΔCash_***t***_*				0.470^**^
				(2.02)
*OC_***t***_*		−0.014	−0.007	−0.005
		(−1.29)	(−0.73)	(−0.58)
*Ind_***t***_*			0.000	
			(0.39)	
*Pro_***t***_*				−0.002
				(−0.33)
*RD*_*t*−1_	2.324^***^	2.577^***^	2.566^***^	2.628^***^
	(6.87)	(7.67)	(7.19)	(7.36)
${\left(RD_{t-1}\right)}^{2}$	−18.015^***^	−20.413^***^	−20.122^***^	−20.807^***^
	(−5.19)	(−5.98)	(−5.68)	(−5.81)
*Stki_***t***_*	−0.050^**^	−0.068^***^	−0.059^**^	−0.057^**^
	(−2.45)	(−2.89)	(−2.47)	(−2.44)
*Stki_*t*−1_*	0.011	0.011	0.012	0.017^*^
	(1.13)	(1.02)	(1.06)	(1.79)
*Loan_***t***_*	−0.002	−0.003	−0.006	−0.005
	(−0.18)	(−0.21)	(−0.47)	(−0.44)
*Loan_*t*−1_*	0.018^*^	0.018	0.022^*^	0.015
	(1.88)	(1.60)	(1.88)	(1.44)
*CF_***t***_*	0.044^*^	0.040	0.044	0.032
	(1.78)	(1.43)	(1.59)	(1.04)
*CF_*t*−1_*	0.007	0.019	0.012	0.014
	(0.38)	(0.85)	(0.53)	(0.63)
*Growth_*t*−1_*	0.001	0.001	0.001	0.001
	(1.18)	(0.81)	(0.91)	(1.00)
*Size_*t*−1_*	0.003	0.006^**^	0.006^**^	0.007^**^
	(1.00)	(2.05)	(2.22)	(2.38)
*Q_*t*−1_*	−0.000	0.000	−0.000	−0.000
	(−0.36)	(0.06)	(−0.19)	(−0.60)
*Age_*t*−1_*	−0.000	0.000	0.000	−0.000
	(−0.04)	(0.26)	(0.18)	(−0.02)
*Constan_***t***_*	−0.120	−0.201^*^	−0.205^**^	−0.229^**^
	(−1.17)	(−1.91)	(−1.97)	(−2.02)
*Year*	Yes	Yes	Yes	Yes
*Ind*	Yes	Yes	Yes	Yes
N	1365	1365	1365	1365
AR (1)	0.000	0.000	0.000	0.000
AR (2)	0.986	0.898	0.719	0.787
Sargan	0.158	0.145	0.211	0.221

*In parentheses are the z statistical values calculated based on robust standard errors*.

*, **, and *** *Represent significance at the 10, 5, and 1% levels, respectively*.

**Figure 2:**
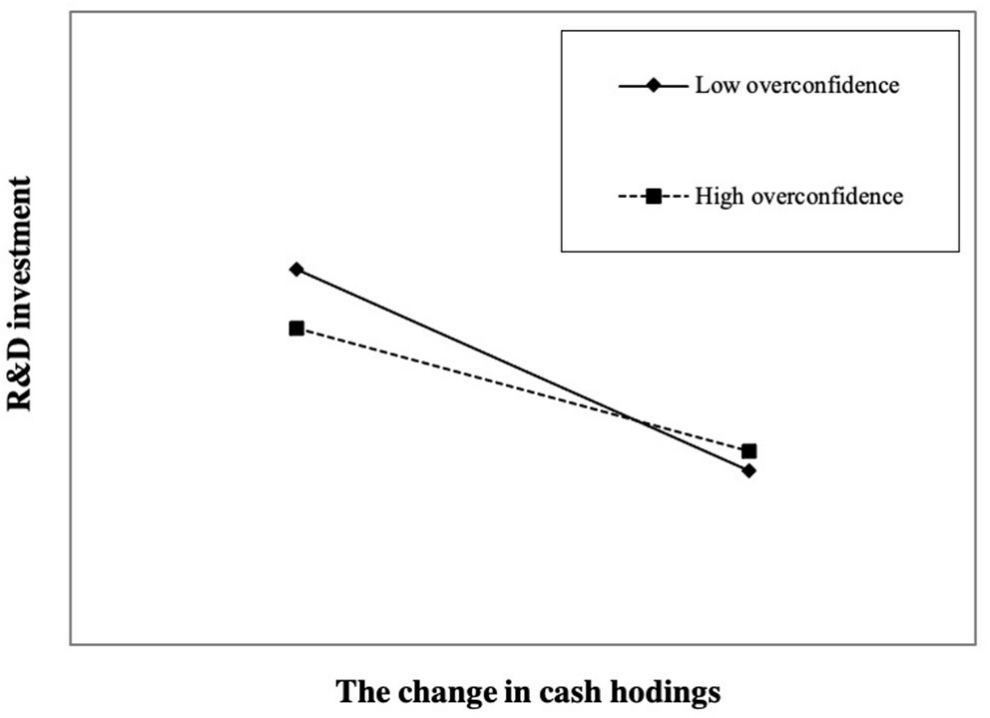
Relationship between CEO overconfidence and R&D smoothing. We plot the interaction effect at different levels of CEO overconfidence according to the result in Table 2, Column (2). A firm with CEO overconfidence equal to one standard deviation above (below) the sample mean is regarded as having high (low) overconfidence. The degree of the negative relationship between cash holding changes and R&D investment represents the level of R&D smoothing; therefore, high overconfidence can weaken the level of R&D smoothing.

The regression results for Equation (2) demonstrate that the estimated coefficient of changes in cash holdings Δ*Cash* β_1_ is −0.118 (*z* = −1.98, *p* < 0.05), and the estimated coefficient of the multiplicative term of overconfidence and changes in cash holdings is 0.256 (*z* = 1.92, *p* < 0.1), which is the opposite sign of the estimated coefficient of Δ*Cash* β_1_. As shown in Figure 2, high CEO overconfidence reduces the negative correlation between changes in cash holdings and R&D investment, which indicates that the R&D smoothing effect is weaker. Therefore, *H1* is supported.

**Figure 3:**
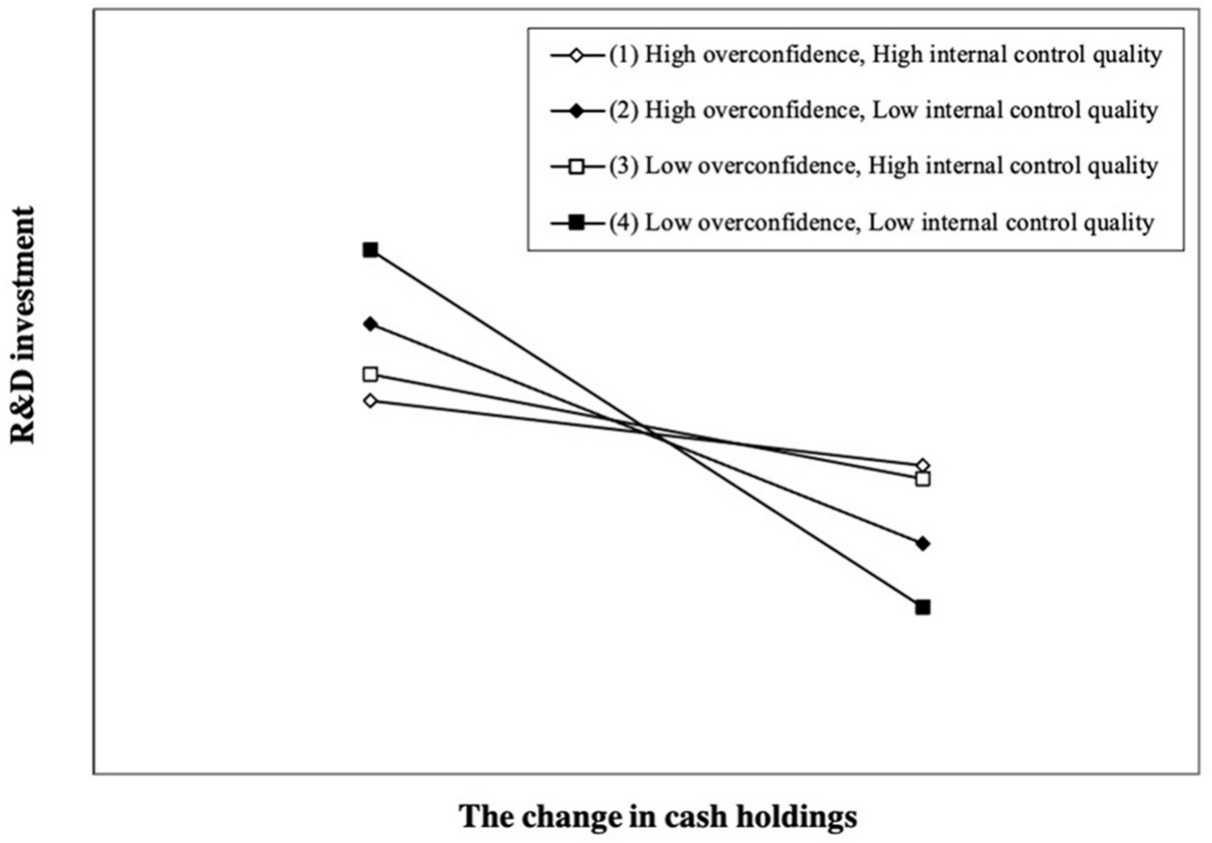
Moderating role of internal control quality. We plot the interaction effect at different levels of internal control quality according to the result in Table 2, Column (3). A firm with internal control quality equal to one standard deviation above (below) the sample mean is regarded as having high (low) internal control quality. A firm with CEO overconfidence equal to one standard deviation above (below) the sample mean is regarded as having high (low) overconfidence. Compared with low internal control quality firms, high internal control quality firms decrease the weakening effect of high CEO overconfidence on R&D smoothing.

Table 2 shows the regression results of Equation (3) regarding the moderating effect of internal control quality on the relationship between CEO overconfidence and R&D smoothing. The estimated coefficient of *OC*×Δ*Cash* β_2_ is 0.480 (*z* = 1.70, *p* < 0.1), and the estimated coefficient of *Ind*×*OC*×Δ*Cash* β_3_ is −0.527 (*z* = −1.78, *p* < 0.1), which is the opposite sign of the estimated coefficient of *OC*×Δ*Cash* β_2_. As shown in Figure 3, high internal control quality reduces the weakening effect of high CEO overconfidence on R&D smoothing. Therefore, *H2* is supported.

**Figure 4:**
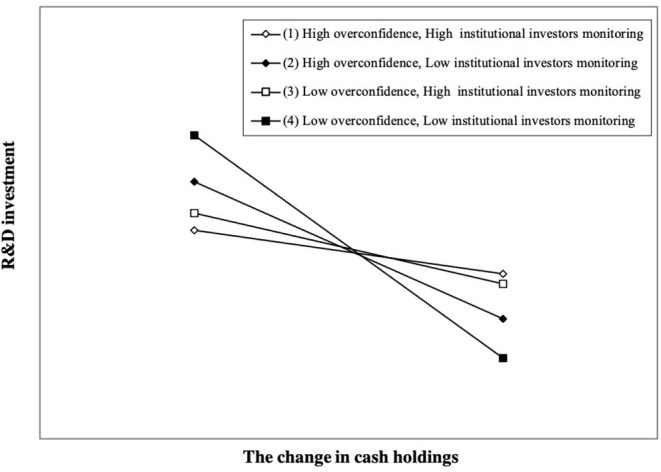
Moderating role of institutional investor monitoring. We plot the interaction effect at different levels of institutional investor monitoring according to the result in Table 2, Column (4). A firm with institutional investor monitoring equal to one standard deviation above (below) the sample mean is regarded as having high (low) institutional investor monitoring. A firm with CEO overconfidence equal to one standard deviation above (below) the sample mean is regarded as having high (low) overconfidence. Compared with firms having low institutional investor monitoring, firms with high institutional investor monitoring decrease the weakening effect of high CEO overconfidence on R&D smoothing.

Furthermore, Table 2 features the regression results of Equation (4) regarding the moderating effect of institutional investor monitoring on the relationship between CEO overconfidence and R&D smoothing. The estimated coefficient of *OC*×Δ*Cash* β_2_ is 0.404 (*z* = 1.67, *p* < 0.1), and the estimated coefficient of *Pro*×*OC*×Δ*Cash* β_3_ is −1.003 (*z* = −1.90, *p* < 0.1), which is the opposite sign of the estimated coefficient of *OC*×Δ*Cash* β_2_. As shown in Figure 4, high institutional investor monitoring reduces the weakening effect of high CEO overconfidence on R&D smoothing. Therefore, *H3* is supported.

### Robustness Check

#### Alternative Measurement of CEO Overconfidence

We modify the measurement of the CEO overconfidence variables to check the robustness of our findings. Specially, we construct a dummy variable for CEO overconfidence. If the sum of the salaries of the top three executives with the highest salaries divided by the sum of the salaries of all executives is greater than the annual median, the value of the overconfidence variable is 1, indicating that the CEO has a relatively high level of overconfidence, and 0 otherwise. The results remain unchanged (Table 3).

**Table 3:** Robustness test results for alternative measurements of CEO overconfidence.

**Variables**	**Equation (1)**	**Equation (2)**	**Equation (3)**	**Equation (4)**
Δ*Cash_*t*_*	−0.024^*^	−0.047^**^	−0.078^**^	−0.054^**^
	(−1.70)	(−2.49)	(−2.30)	(−1.99)
*OC × ΔCash_*t*_*		0.040^**^	0.071^**^	0.052^*^
		(2.22)	(2.19)	(1.79)
*Ind × OC × ΔCash_*t*_*			−0.083^**^	
			(−2.30)	
*Pro × OC × ΔCash_*t*_*				−0.135^**^
				(−2.21)
*Ind × ΔCash_*t*_*			0.084^**^	
			(2.45)	
*Pro × ΔCash_*t*_*				0.128^**^
				(2.24)
*OC_***t***_*		0.000	0.001	0.001
		(0.01)	(0.76)	(0.64)
*Ind_***t***_*			0.000	
			(0.01)	
*Pro_***t***_*				−0.002
				(−0.42)
*RD*_*t*−1_	2.324^***^	2.374^***^	2.492^***^	2.434^***^
	(6.87)	(7.12)	(7.37)	(7.31)
${\left(RD_{t-1}\right)}^{2}$	−18.015^***^	−18.488^***^	−19.530^***^	−18.992^***^
	(−5.19)	(−5.40)	(−5.69)	(−5.54)
*Stki_***t***_*	−0.050^**^	−0.055^***^	−0.056^**^	−0.055^**^
	(−2.45)	(−2.59)	(−2.53)	(−2.53)
*Stki_*t*−1_*	0.011	0.011	0.009	0.018^*^
	(1.13)	(1.00)	(0.83)	(1.85)
*Loan_***t***_*	−0.002	−0.003	−0.003	−0.003
	(−0.18)	(−0.24)	(−0.24)	(−0.30)
*Loan_*t*−1_*	0.018^*^	0.021^**^	0.020^*^	0.015
	(1.88)	(1.97)	(1.90)	(1.55)
*CF_***t***_*	0.044^*^	0.043^*^	0.046^*^	0.038
	(1.78)	(1.66)	(1.75)	(1.41)
*CF_*t*−1_*	0.007	0.011	0.009	0.011
	(0.38)	(0.58)	(0.47)	(0.56)
*Growth_*t*−1_*	0.001	0.001	0.001	0.001
	(1.18)	(0.99)	(0.73)	(1.11)
*Size_*t*−1_*	0.003	0.003	0.005	0.005^*^
	(1.00)	(1.21)	(1.62)	(1.94)
*Q_*t*−1_*	−0.000	−0.000	−0.000	−0.000
	(−0.36)	(−0.23)	(−0.37)	(−0.72)
*Age_*t*−1_*	−0.000	0.000	−0.000	−0.000
	(−0.04)	(0.06)	(−0.12)	(−0.18)
*Constan_***t***_*	−0.120	−0.137	−0.153	−0.183^*^
	(−1.17)	(−1.35)	(−1.53)	(−1.80)
*Year*	Yes	Yes	Yes	Yes
*Ind*	Yes	Yes	Yes	Yes
N	1365	1365	1365	1365
AR (1)	0.000	0.000	0.000	0.000
AR (2)	0.986	0.322	0.830	0.943
Sargan	0.158	0.169	0.209	0.202

*In parentheses are the z statistical values calculated based on robust standard errors*.

*, **, and *** *Represent significance at the 10, 5, and 1% levels, respectively*.

#### Alternative Estimation Strategies

Our findings are robust to a number of instrument sets. We adjust the instrument set of the core variable (Δ*Cash*_*i*;*t*_) to include levels dated *t*−*2* to *t*−*3* for the regression in differences, and lagged differences dated for the regression in levels. The results remain unchanged (Table 4).

**Table 4:** Robustness test results for alternative estimation strategies.

**Variables**	**Equation (1)**	**Equation (2)**	**Equation (3)**	**Equation (4)**
Δ*Cash_*t*_*	−0.027^*^	−0.128^**^	−0.269^*^	−0.204^*^
	(−1.74)	(−2.09)	(−1.90)	(−1.90)
*OC × ΔCash_*t*_*		0.276^**^	0.571^*^	0.433^*^
		(2.01)	(1.86)	(1.74)
*Ind × OC × ΔCash_*t*_*			−0.617^*^	
			(−1.91)	
*Pro × OC × ΔCash_*t*_*				−1.058^*^
				(−1.96)
*Ind × ΔCash_***t***_*			0.288^*^	
			(1.94)	
*Pro × ΔCash_***t***_*				0.496^**^
				(2.09)
*OC_***t***_*		−0.014	−0.007	−0.005
		(−1.26)	(−0.72)	(−0.52)
*Ind_***t***_*			0.000	
			(0.36)	
*Pro_***t***_*				−0.001
				(−0.29)
*RD*_*t*−1_	2.326^***^	2.620^***^	2.616^***^	2.669^***^
	(6.66)	(7.73)	(7.16)	(7.35)
${\left(RD_{t-1}\right)}^{2}$	−18.061^***^	−20.836^***^	−20.589^***^	−21.212^***^
	(−5.12)	(−6.08)	(−5.71)	(−5.86)
*Stki_***t***_*	−0.047^**^	−0.065^***^	−0.054^**^	−0.053^**^
	(−2.22)	(−2.68)	(−2.22)	(−2.23)
*Stki_*t*−1_*	0.009	0.010	0.009	0.016
	(0.91)	(0.95)	(0.80)	(1.63)
*Loan_***t***_*	0.000	−0.000	−0.004	−0.004
	(0.00)	(−0.01)	(−0.26)	(−0.28)
*Loan_*t*−1_*	0.017^*^	0.016	0.021^*^	0.013
	(1.70)	(1.42)	(1.69)	(1.24)
*CF_***t***_*	0.052^**^	0.047	0.053^*^	0.040
	(2.05)	(1.63)	(1.81)	(1.26)
*CF_*t*−1_*	0.013	0.025	0.019	0.020
	(0.67)	(1.11)	(0.81)	(0.89)
*Growth_*t*−1_*	0.001	0.001	0.001	0.001
	(1.17)	(0.80)	(0.87)	(0.95)
*Size_*t*−1_*	0.003	0.006^**^	0.007^**^	0.007^**^
	(0.96)	(2.14)	(2.31)	(2.53)
*Q_*t*−1_*	−0.000	0.000	−0.000	−0.000
	(−0.41)	(0.06)	(−0.22)	(−0.63)
*Age_*t*−1_*	−0.000	0.000	0.000	0.000
	(−0.02)	(0.31)	(0.23)	(0.01)
*Constan_***t***_*	−0.124	−0.216^**^	−0.222^**^	−0.248^**^
	(−1.17)	(−1.99)	(−2.05)	(−2.11)
*Year*	Yes	Yes	Yes	Yes
*Ind*	Yes	Yes	Yes	Yes
N	1,365	1,365	1,365	1,365
AR (1)	0.000	0.000	0.000	0.000
AR (2)	0.875	0.974	0.759	0.685
Sargan	0.277	0.279	0.376	0.226

*In parentheses are the z statistical values calculated based on robust standard errors*.

*, **, and *** *represent significance at the 10, 5, and 1% levels, respectively*.

#### Controlling for Additional Investment

As the investment regressions may also by affected by the TBEFs' short-term investment ability, we also use the additional investment (*Caper*_*t*−1_) as a control variable, which is measured by the ratio of Cash paid for purchase and construction of fixed assets, intangible assets, and other long-term assets divided by the book value of total assets at the end of the period *t*−*1*. Table 5 lists the results of controlling for TBEFs' new investment and the results remain unchanged.

**Table 5:** Robustness test results for controlling TBEFs' additional investment.

**Variables**	**Equation (1)**	**Equation (2)**	**Equation (3)**	**Equation (4)**
Δ*Cash_*t*_*	−0.025^*^	−0.118^**^	−0.218^*^	−0.187^*^
	(−1.79)	(−1.98)	(−1.70)	(−1.80)
*OC × ΔCash_*t*_*		0.255^*^	0.464^*^	0.397^*^
		(1.92)	(1.68)	(1.66)
*Ind × OC × ΔCash_*t*_*			−0.511^*^	
			(−1.75)	
*Pro × OC × ΔCash_*t*_*				−0.988^*^
				(−1.90)
*Ind × ΔCash_***t***_*			0.238^*^	
			(1.78)	
*Pro × ΔCash_***t***_*				0.464^**^
				(2.02)
*OC_***t***_*		−0.014	−0.007	−0.005
		(−1.29)	(−0.71)	(−0.57)
*Ind_***t***_*			0.000	
			(0.39)	
*Pro_***t***_*				−0.001
				(−0.28)
*RD*_*t*−1_	2.314^***^	2.566^***^	2.551^***^	2.613^***^
	(6.88)	(7.72)	(7.27)	(7.41)
${\left(RD_{t-1}\right)}^{2}$	−17.926^***^	−20.292^***^	−19.961^***^	−20.637^***^
	(−5.22)	(−6.03)	(−5.76)	(−5.86)
*Stki_***t***_*	−0.051^**^	−0.069^***^	−0.059^**^	−0.057^**^
	(−2.47)	(−2.91)	(−2.51)	(−2.47)
*Stki_*t*−1_*	0.011	0.011	0.011	0.017^*^
	(1.12)	(0.99)	(1.05)	(1.74)
*Loan_***t***_*	−0.002	−0.002	−0.006	−0.005
	(−0.16)	(−0.16)	(−0.43)	(−0.37)
*Loan_*t*−1_*	0.018^*^	0.018	0.021^*^	0.014
	(1.86)	(1.56)	(1.83)	(1.39)
*CF_***t***_*	0.045^*^	0.042	0.046^*^	0.034
	(1.84)	(1.50)	(1.69)	(1.11)
*CF_*t*−1_*	0.006	0.016	0.009	0.011
	(0.31)	(0.77)	(0.42)	(0.52)
*Growth_*t*−1_*	0.001	0.001	0.001	0.001
	(1.19)	(0.79)	(0.89)	(0.98)
*Size_*t*−1_*	0.003	0.006^**^	0.006^**^	0.007^**^
	(0.92)	(2.02)	(2.17)	(2.36)
*Q_*t*−1_*	−0.000	0.000	−0.000	−0.000
	(−0.35)	(0.09)	(−0.16)	(−0.57)
*Age_*t*−1_*	−0.000	0.000	0.000	−0.000
	(−0.02)	(0.26)	(0.18)	(−0.02)
*Caper_*t*−1_*	0.002	−0.005	−0.003	−0.007
	(0.14)	(−0.38)	(−0.27)	(−0.54)
*Constan_***t***_*	−0.115	−0.199^*^	−0.201^*^	−0.227^**^
	(−1.13)	(−1.90)	(−1.95)	(−2.01)
*Year*	Yes	Yes	Yes	Yes
*Ind*	Yes	Yes	Yes	Yes
N	1365	1365	1365	1365
AR (1)	0.000	0.000	0.000	0.000
AR (2)	0.987	0.894	0.729	0.799
Sargan	0.171	0.175	0.200	0.223

*In parentheses are the z statistical values calculated based on robust standard errors*.

*, **, and *** *Represent significance at the 10, 5, and 1% levels, respectively*.

## Discussion

### Conclusion

In this study, we use a sample of non-financial firms from China's Growth Enterprises Market between 2013 and 2020 to investigate how CEO overconfidence affects firms' R&D smoothing behavior. We then examine the moderating effects of two types of corporate governance mechanisms (i.e., internal control quality and institutional investor monitoring) on CEO overconfidence and R&D smoothing. The results show that CEO overconfidence has a significant negative impact on TBEFs' R&D smoothing behavior. However, improving these firms' internal and external governance mechanisms can mitigate the negative association between CEO overconfidence and R&D smoothing.

### Theoretical and Practical Implications

This study offers several theoretical implications. First, we add to the body of research on the antecedents of TBEFs' R&D smoothing from the upper echelons theoretical perspective, which provides an understanding of the differences in TBEFs' intertemporal dynamic adjustment behavior regarding R&D investments. The impact on R&D smoothing from organizational and contingent perspectives has been explored in the literature (Brown and Petersen, 2011; Shin and Kim, 2011; Liu et al., 2021; Yang et al., 2021). However, CEOs are usually the key decision-makers at firms. By focusing on the CEO-level factors of R&D smoothing, this study adds to the literature on the nexus of the CEO and dynamic, intertemporal R&D investment decisions. This study also responds to calls to investigate the microfoundations of firms' strategic decisions (Yu et al., 2022b).

Our findings further enrich the body of research on the economic consequences of CEO overconfidence. Extending the results of prior studies focusing on the static characteristics of R&D investment (Hirshleifer et al., 2012), we emphasize the long-term dynamic adjustment process of R&D investments and find that CEO overconfidence inhibits R&D smoothing behavior, which represents an intertemporal R&D investment decision. Our results show that firms' current level of R&D investment may yield unfavorable results in the future because of CEOs' unwillingness to engage in measures like R&D smoothing to deal with intertemporal uncertainty and mitigate any future issues. While TBEFs must consider uncertainty (Yu et al., 2018), their unwillingness to engage in R&D smoothing will likely hinder their steady progress in future R&D activities.

Furthermore, we extend the literature on internal control in the context of overconfident CEOs' investment behaviors by considering intertemporal investment behaviors. We provide new insights into the effects of internal control on overconfident CEOs' intertemporal investment decision-making behavior from the perspective of internal governance. Kolasinski and Li (2013) demonstrate that internal control has a negative effect on overconfident CEOs' mergers and acquisitions behavior. Our results show that high-quality internal control can mitigate the inhibitory effect of overconfident CEOs on their R&D smoothing behavior. In other words, improving internal control quality can help firms maintain their R&D activities. This finding reinforces the key role of internal control in corporate governance.

Finally, we add to the literature on the effects of institutional investor monitoring. Earlier studies noted that institutional investors could influence various corporate decisions (Chung et al., 2002; Ferreira et al., 2010; Tai et al., 2014). In extending this stream of literature, our findings demonstrate that a higher level of institutional investor monitoring can control the inhibitory effect of overconfident CEOs on R&D smoothing, which reflects the importance of institutional investors' role in governance.

This study also has several practical implications. First, environmental uncertainty has brought great challenges to firm innovation (Yu et al., 2020; Lou et al., 2022). Therefore, uncertainties could lead to the unsustainability of innovative activities. CEOs' strategic choices matter to TBEFs' innovative activities (Wang X., et al., 2020); therefore, we suggest that CEOs of TBEFs should avoid excessive overconfidence in themselves to maintain the level of R&D smoothing of their firms with a view toward increasing their firms' level of preparedness regarding future uncertainties. Second, compared with mature companies, TBEFs feature lower levels of internal control. However, internal control is a key component in quelling the behavior of overconfident CEOs. Therefore, we suggest that TBEFs, especially those with overconfident CEOs, should focus on internal control. Third, compared with mature companies, institutional investors in TBEFs exert greater control over the CEOs' decisions, especially in situations where CEOs exhibit high levels of overconfidence (Yu et al., 2022a). TBEFs should focus on improving internal control quality and actively improve their institutional investor monitoring to ensure the long-term continuity of their R&D activities.

### Limitations and Opportunities

Our study has some limitations that offer opportunities for the nexus of the CEO and dynamic, intertemporal R&D investment decisions. First, we assume that the external environment will not affect overconfident CEOs' intertemporal decision-making behavior when we examine the relationship between CEO overconfidence and R&D smoothing behavior. There is no evidence for how overconfident CEOs affect R&D smoothing behavior when their TBEF exists in a dynamic external environment. However, there are opportunities for adding additional business environment factors (Zhao et al., 2019; Wang B., et al., 2020) into the theoretical model of the relationship between CEO overconfidence and R&D smoothing to expand the generalizability of the results of our study. We also assume that CEOs' overconfidence is a personal characteristic that does not change over time. As our study focuses on the differences in overconfidence levels between CEOs across a variety of companies, we note that some CEOs are more likely to be overconfident than others. Therefore, our study does not consider changes in CEOs' personal overconfidence levels over time. In addition, in the measurement of CEO overconfidence, there is a certain deviation because of the data. These factors provide opportunities for improving the method for measuring CEO overconfidence. Finally, this study only understands the micro-foundations of R&D smoothing strategic decision-making behavior from the perspective of CEO overconfidence. Future studies could explore the use of new technologies, such as physiological and neuroscientific tools (Yu et al., 2022b), to further understand the effect of CEOs' emotions on their R&D smoothing decision-marking processes.

## Data Availability Statement

The raw data supporting the conclusions of this article will be made available by the authors, without undue reservation.

## Author Contributions

All authors listed have made a substantial, direct, and intellectual contribution to the work and approved it for publication.

## Funding

The research was supported by the Natural Science Foundation of China (71972126, 71772117), Innovation Program of Shanghai Municipal Education Commission (2019-01-07-00-09-E00078).

## Conflict of Interest

The authors declare that the research was conducted in the absence of any commercial or financial relationships that could be construed as a potential conflict of interest.

## Publisher's Note

All claims expressed in this article are solely those of the authors and do not necessarily represent those of their affiliated organizations, or those of the publisher, the editors and the reviewers. Any product that may be evaluated in this article, or claim that may be made by its manufacturer, is not guaranteed or endorsed by the publisher.

## Appendix

**Appendix 1:** Variable definitions.

**Variables**	**Names**	**Definitions**
*RD_t_*	R&D investment	R&D expenditure divided by total assets at the end of period *t*.
Δ*Cash_t_*	The change in cash holding	The change in monetary funds and trading financial assets from the beginning to the end of period *t*, divided by the book value of total assets at the beginning of period *t*.
*OC_t_*	CEO overconfidence	The ratio of the sum of the salaries of the top three executives with the highest salaries divided by the sum of the salaries of all executives.
*Ind_t_*	Internal control quality	Dummy variable. If China Listed Firms' Internal Control Index is greater than the industry annual median, the value of internal control quality is 1, and 0 otherwise.
*Pro_t_*	Institutional investor monitoring	Institutional investors' shareholding ratio.
*Stki_t_*	Equity financing	Cash received from absorbing equity investments divided by the book value of total assets at the end of period *t*.
*Loan_t_*	Debt financing	Cash received from issuing bonds and obtaining loans divided by the book value of total assets at the end of period *t*.
*CF_t_*	Cash flow	Net cash flow from operating activities divided by the book value of total assets at the end of period *t*.
*Growth_t−−1_*	Growth rate of operating revenue	The change in operating revenue between the beginning and end of period *t* divided by the operating revenue at the end of period *t–1*.
*Size_t−−1_*	Firm size	Log of the book value of total assets at the end of period *t–1*.
*Age_t−−1_*	Firm age	Difference between the period *t–1* and the registration year.
*Q_t−−1_*	Market-to-book ratio	Market value of assets in period *t–1* divided by the book value of total assets in period *t–1*.
*Caper_t−−1_*	Additional investment	Cash paid for purchase and construction of fixed assets, intangible assets, and other long-term assets divided by the book value of total assets at the end of the period *t–1*.

*The following are the brief description of figures used in this manuscript*.
